# Prescription of renin–angiotensin system blockers and risk of acute kidney injury: a population-based cohort study

**DOI:** 10.1136/bmjopen-2016-012690

**Published:** 2016-12-21

**Authors:** Kathryn E Mansfield, Dorothea Nitsch, Liam Smeeth, Krishnan Bhaskaran, Laurie A Tomlinson

**Affiliations:** Department of Non-Communicable Disease Epidemiology, London School of Hygiene and Tropical Medicine, London, UK

**Keywords:** acute kidney injury, angiotensin-converting enzyme inhibitors, angiotensin receptor antagonists, cohort study, renin-angiotensin system

## Abstract

**Objective:**

To investigate whether there is an association between use of ACE inhibitors (ACEI) and angiotensin receptor blockers (ARB) and risk of acute kidney injury (AKI).

**Study design:**

We conducted a new-user cohort study of the rate of AKI among users of common antihypertensives.

**Setting:**

UK primary care practices contributing to the Clinical Practice Research Datalink (CPRD) eligible for linkage to hospital records data from the Hospital Episode Statistics (HES) database between April 1997 and March 2014.

**Participants:**

New users of antihypertensives: ACEI/ARB, β-blockers, calcium channel blockers and thiazide diuretics.

**Outcomes:**

The outcome was first episode of AKI. We estimated incidence rate ratio (RR) for AKI during time exposed to ACEI/ARB compared to time unexposed, adjusting for age, sex, comorbidities, use of other antihypertensive drugs and calendar period using Poisson regression. Covariates were time updated.

**Results:**

Among 570 445 participants, 303 761 were prescribed ACEI/ARB with a mean follow-up of 4.1 years. The adjusted RR of AKI during time exposed to ACEI/ARB compared to time unexposed was 1.12 (95% CI 1.07 to 1.17). This relative risk varied depending on absolute risk of AKI, with lower or no increased relative risk from the drugs among those at greatest absolute risk. For example, among people with stage 4 chronic kidney disease (who had 6.69 (95% CI 5.57 to 8.03) times higher rate of AKI compared to those without chronic kidney disease), the adjusted RR of AKI during time exposed to ACEI/ARB compared to time unexposed was 0.66 (95% CI 0.44 to 0.97) in contrast to 1.17 (95% CI 1.09 to 1.25) among people without chronic kidney disease.

**Conclusions:**

Treatment with ACEI/ARB is associated with only a small increase in AKI risk while individual patient characteristics are much more strongly associated with the rate of AKI. The degree of increased risk varies between patient groups.

**Strengths and limitations of this study**

- This is the largest study of this topic to date; it examines an inclusive population-based cohort and reflects routine clinical use of these medications.
- By comparing ACE inhibitors/angiotensin receptor blockers use to use of other antihypertensives, we were able to reduce confounding by indication compared to previous case–control studies.
- We were able to clearly define and adjust for covariates, including renal function, prior to starting the medication. The time-updated analysis reduced residual confounding, while restriction to only incident users reduced adherence bias.
- However, there are a number of important limitations. Our assessment of drug exposure was based on prescriptions so we cannot be certain that people prescribed the drug were taking the medication.
- We did not have inpatient biochemical data so could only use International Classification of Diseases 10th revision (ICD-10) coding to define acute kidney injury (AKI). Therefore, we have captured only a proportion of the cases defined by current biochemical definitions of AKI, although this includes a greater proportion of more severe cases.

## Introduction

Acute kidney injury (AKI) is a sudden decline in renal function, affecting up to 20% of people admitted to hospital, and is strongly associated with increased mortality and longer duration of hospital stay.^1^ Prevention and better management of patients with AKI is the focus of national programmes^2^ and global campaigns.^3^

It is strongly believed that ACE inhibitors (ACEI) and angiotensin receptor blockers (ARB) are associated with development of AKI, particularly during acute illness. ACEI/ARBs cause preferential vasodilation of the kidney's efferent arterioles (the small blood vessels that leave the kidney glomeruli) thereby reducing kidney filtration pressure for a given systemic blood pressure. During severe hypovolaemia or hypotension (eg, due to volume depletion in acute illness), this reduction of efferent vascular tone leads to reduced glomerular filtration and potentially AKI.^4^ While biologically plausible, evidence to support the belief that ACEI/ARB use causes AKI is limited. The incidence of AKI in randomised controlled trials of ACEI and ARB compared to placebo is poorly described due to variable definitions or absent reporting of kidney-related adverse events.^5^ Previous observational studies have compared the risk of AKI in patients using ACEI/ARB alone to the risks among ACEI/ARB users also taking diuretics and/or non-steroidal anti-inflammatory drugs (NSAIDs),^6–8^ or with ACEI/ARB alone under specific circumstances.^9–11^ However, the risk of AKI in patients taking ACEI or ARB alone compared to other comparator drugs has not been examined in a population cohort using individual patient data. In contrast, high-quality evidence from randomised trials of increased risk of AKI associated with dual prescription of ACEI and ARB^12^ ^13^ compared to single agent therapy has led to a restriction on the use of these drugs in combination.^14^

Despite this limited evidence, there is a growing consensus that ACEI/ARB should be withheld during acute illness.^15^ ^16^ Guidelines for patients to self-manage medications linked to AKI during these situations, known as ‘sick day rules’, are being widely introduced.^17^ Therefore, we aimed to investigate the association between AKI and the use of ACEI/ARB in a large population-based cohort study of people starting treatment with commonly used antihypertensive drugs (ACEI/ARB, β blockers, calcium channel blockers, thiazide diuretics). We chose to compare new users of different classes of antihypertensive drugs to reduce confounding by indication.

## Methods

### Study design and setting

We undertook a cohort study using the UK Clinical Practice Research Datalink (CPRD) and linked hospital record data from the Hospital Episode Statistics (HES) database. CPRD is a database of routinely collected primary care electronic health record data from 7% of the UK population.^18^ Included patients are largely representative of the UK population.^18–20^ HES records cover all admissions for NHS funded patients treated in either English NHS trusts or by independent providers.^21^ Fifty-eight per cent of general practices included in CPRD are linked to HES data (representing 75% of English practices).^18^ We used only fully linked data from CPRD and HES to ensure that all participants had complete data regarding the exposure (antihypertensive prescribing in primary care) and the outcome (hospital admission with AKI). The study period was from 1 April 1997 to 31 March 2014, the latest date for which there is HES data linkage to CPRD. This study was approved by the LSHTM Research Ethics Committee (reference 6536) and by the CPRD independent scientific advisory committee (ISAC protocol number: 14-208).

### Participants, exposures and outcomes

To minimise confounding by indication, rather than comparing ACEI/ARB users to otherwise healthy individuals, we identified a cohort of new users of drugs that were prescribed for similar indications to ACEI/ARB. We developed a cohort of all HES-linked CPRD patients aged 18 years or older who were new users of antihypertensive drugs (ACEI/ARB, β blockers, calcium channel blockers or thiazide diuretics) during the study period. The primary exposure was use of ACEI/ARB, and other drugs were treated as potential confounders. To ensure that we had reliable measures of drug use and baseline covariates, we required that all participants had at least 1 year of continuous registration in CPRD before the first recorded antihypertensive drug prescription. We calculated the length of each prescription using the quantity of medication prescribed and the daily dose recorded, excluding patients for whom dosing information was inadequate to obtain a robust duration of exposure. Exposure to medications was assumed to start on the date of the prescription. We identified continuous courses of therapy by allowing for a 60-day gap between the end date of one prescription and the start of the next consecutive prescription (to allow for stock piling of medications).

Drug exposure status was time updated based on continuous courses of therapy. We defined exposure status using four time-varying, binary indicator variables to indicate exposure to each antihypertensive, with exposure status ‘switching on’ when an individual was prescribed a drug and ‘off’ when their prescription ended (example scenarios illustrating the assignment of indicator variables are included in online supplementary text S1 and figure S1). This allowed us to maximise the available follow-up time, control for exposure to other antihypertensives, allowed drug combinations to be investigated through interaction terms and more closely modelled real life prescribing patterns.

10.1136/bmjopen-2016-012690.supp1 supplementary data

Follow-up started at first prescription for the first of any of the antihypertensive drugs and ended at either occurrence of the outcome or the earliest of (1) end of final prescription; (2) death; (3) left GP practice; (4) last data collection or (5) diagnosis of end-stage renal disease (ESRD) (see online supplementary text S2). We excluded patients with ESRD prior to cohort entry.

We defined the outcome as the first episode of AKI identified within 28 days of the start of a hospital admission identified using ICD-10 morbidity coding in HES (see online supplementary table S1), to capture cases of AKI that were present at hospital admission but may have not been immediately diagnosed, without excluding cases that resulted in a prolonged admission. The actual number of AKI cases is likely to be higher than that captured by ICD-10 coding as less severe cases may not result in hospitalisation or may not be coded in hospital records.

### Covariates

Owing to the complex and overlapping potential risk factors for AKI, we used a directed acyclic graph (DAG) approach to visualise our a priori assumptions about the potential biological mechanisms between exposure and outcome and to guide adjustment for confounding in sequentially adjusted regression models (see online supplementary figure S2).^22^ By asking researchers to produce an illustration of the a priori paths between exposure, outcome and potential confounders, causal diagrams offer a “starting point for identifying variables that must be measured and controlled [for] to obtain unconfounded effect estimates”.^23^ We identified potential confounders based on clinical knowledge and previous research investigating predictors of AKI.^6^ ^7^ ^9^ ^10^

We adjusted for baseline chronic kidney disease (CKD) stage, established by calculating estimated glomerular filtration rate (eGFR) using the Chronic Kidney Disease Epidemiology Collaboration (CKD-EPI) equation.^24^ We used serum creatinine results recorded in the 12 months before first prescription to calculate eGFR, using either the highest eGFR from the most recent two serum creatinine results, separated by a minimum of 3 months or, if only one creatinine result was available, the single most recent serum creatinine recorded prior to first prescription. Serum creatinine measurements were not routinely isotope-dilution mass spectrometry (IDMS)-standardised until 2013. We therefore assumed that all creatinine results were unstandardised and multiplied results with a correction factor of 0.95 before calculating eGFR without regard to ethnicity.^25^ To avoid selection bias, we included an absent CKD category for those with no recorded serum creatinine result in the 12 months prior to first antihypertensive prescription.

Other chronic comorbidities included as confounders were as follows: diabetes mellitus, hypertension, cardiac failure, cardiac arrhythmia and ischaemic heart disease, identified from CPRD and HES data. In regression analyses, these comorbidities were recorded as time-varying variables representing ‘ever diagnosed,’ whose status changed with the first recorded code for each specific condition. Age group was entered as a time-updating variable. We adjusted for time-varying exposure to loop and potassium-sparing diuretics in addition to antihypertensive drugs.^7^

We used existing morbidity code lists and algorithms for ethnicity,^19^ smoking status, alcohol intake, body mass index (BMI)^20^ and chronic comorbidities.^26–30^ Socioeconomic status was defined using quintiles of index of multiple deprivation scores for 2004.

We included calendar period as a covariate to adjust for the many changes in clinical, diagnostic and administrative practices over the study period that may influence the measurement of baseline renal function and number of reported AKI cases.

### Statistical analysis

When variables (such as drug exposure, age and comorbidities) did not remain constant over time, we defined them as time-varying variables. We did this by splitting the data for each study subject into several observations, each observation started on the date of a change in that subject's status (eg, the prescription of a new drug, the diagnosis of a new comorbidity or a change in age). In the main analysis, we classified exposure status using a time-varying binary indicator variable for person-time prescribed an ACEI/ARB. Rather than comparing a group of individual patients prescribed a particular class of drugs to another group prescribed a different class, we compared person-time taking one drug to person-time taking another. To avoid immortal time bias, we excluded all time when patients were not taking any antihypertensive drugs. We estimated RRs associated with time exposed to antihypertensive treatment including an ACEI/ARB, compared to time exposed to antihypertensive treatment that did not include an ACEI/ARB, adjusting for potential confounders using Poisson regression. We used robust SEs to account for clustering by general practice. We initially adjusted for age and sex only and then fitted an adjusted model including DAG-informed time-varying confounders (age, sex, chronic comorbidities, other antihypertensive drugs, loop and potassium-sparing diuretics and calendar period). Further adjustments were for smoking, alcohol, BMI and socioeconomic status. All data management and analyses were performed using Stata V.13 (StataCorp, Texas, USA). We have made code lists for all covariates available in online repository at: https://clinicalcodes.rss.mhs.man.ac.uk/.^31^

### Sensitivity analyses

To determine the impact of including individuals with unknown baseline renal function, we repeated the main analysis in the subgroup of the cohort with known baseline renal function. Next, we repeated the main analysis in new entrants to the cohort, who had ethnicity recorded in CPRD or HES, after 2006 when recording of ethnicity was rewarded in primary care leading to improvements in CPRD data completeness.^19^ We included ethnicity in the equation used to calculate eGFR and as a covariate in the analysis. Finally, we tested the robustness of the definition of AKI in a range of sensitivity analyses including limiting the defining ICD-10 code to just N17, which has a high positive predictive value for AKI.^32^

### Additional analyses

We conducted three additional analyses. First, we investigated the impact of including interaction terms between treatment with loop diuretics and, separately, potassium-sparing diuretics and ACEI/ARB—as concurrent use of ACEI/ARB and diuretics has been linked to increased risk of AKI.^6^ ^7^ In our second additional analysis, renal function was time updated to examine how the relationship between AKI and ACEI/ARB exposure was related to renal function at the time that AKI occurred, rather than at entry to the cohort. To minimise misclassification of CKD stage by renal function measured during an AKI episode, we excluded all measurements of kidney function that occurred within 1 week of an admission with AKI.^33^ Finally, we investigated whether there was any difference in the rate of AKI during time exposed to ACEI compared to ARB and during combination therapy.^12^ ^13^

## Results

### Study population and baseline characteristics

**Figure 1:**
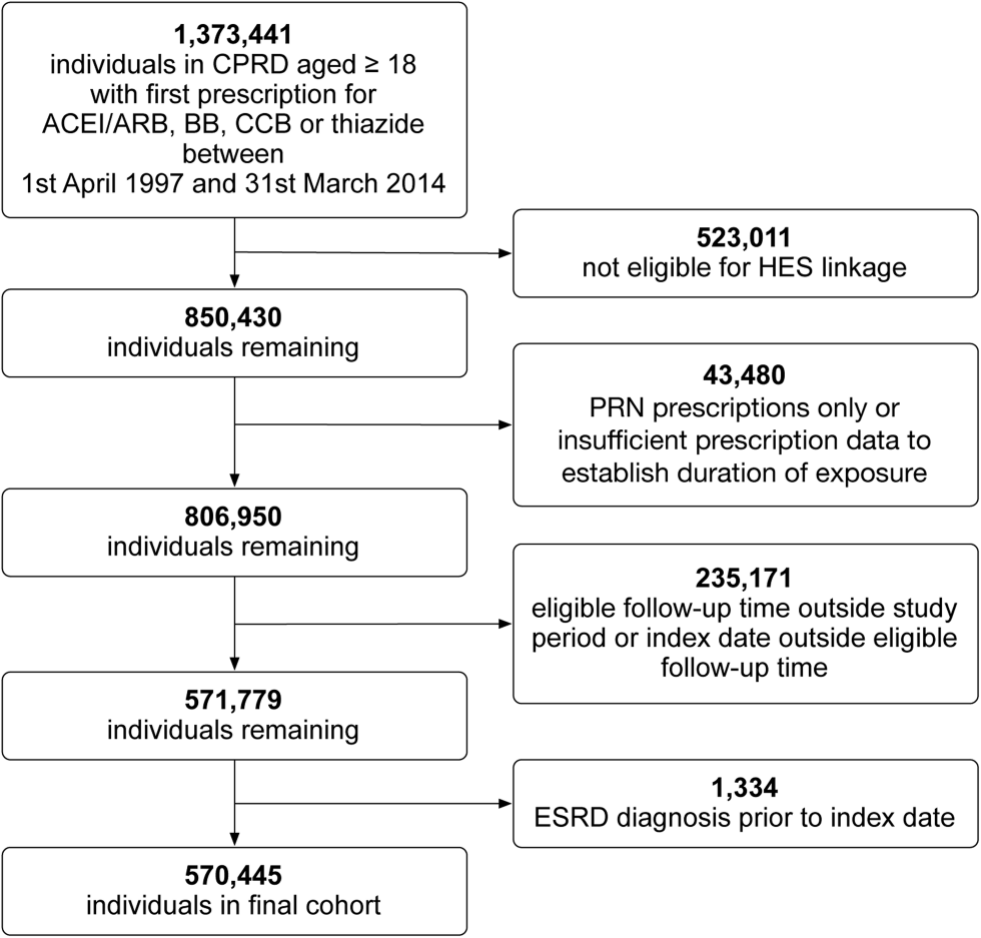
Flow diagram showing the creation of the cohort and reasons for exclusion. ACEI/ARB, ACE inhibitors inhibitor/angiotensin receptor blocker; BB, β blocker; CCB, calcium channel blocker; CPRD, Clinical Practice Research Datalink; HES, Hospital Episode Statistics; ESRD, end-stage renal disease.

Of 1 373 441 individuals aged 18 years or older with a new prescription for an ACEI/ARB, β blocker, calcium channel blocker or thiazide diuretic identified in the CPRD between April 1997 and March 2014, 570 445 were included in the final cohort (figure 1). Of these, 303 763 (53%) were prescribed an ACEI/ARB during follow-up. Total follow-up time for the whole cohort was over 2.3 million person years and 56% (1 320 001/2 345 098) of that was time exposed to ACEI/ARB. Follow-up ended a mean of 4.1 years (SD 4.1) after first antihypertensive drug prescription. A total of 14 907 people developed AKI. The characteristics of the overall cohort and the cohort during time exposed to antihypertensive treatment regimens that either included or excluded an ACEI/ARB are presented in table 1. Those exposed to ACEI/ARB were more likely to be men with cardiac comorbidities and to have had renal function measured prior to starting an antihypertensive. Fifty-three per cent of time exposed to antihypertensive treatment including an ACEI/ARB was between 2009 and 2014 compared to 38% of time exposed to antihypertensive treatment excluding an ACEI/ARB.

**Table 1:** Person-time under follow-up broken down by patient-level characteristics and ACEI/ARB exposure status

	Whole cohort	Cohort during time exposed to antihypertensive treatment including an ACEI/ARB	Cohort during time exposed to antihypertensive treatment excluding an ACEI/ARB
Total person years at risk	2 345 098	1 320 001*	1 025 097*
Median person years at risk (IQR)	2.8 (0.4–7)	3.6 (1.1–6.9)	0.8 (0.2–3.4)
Range of person years at risk	0.0–17.0	0.0–17.0	0.0–17.0
AKI			
Number of events	14 907	10 157	4750
Sex			
Female	1 152 897 (49.2)	577 957 (43.8)	574 940 (56.1)
Age (years)			
18–44	151 515 (6.5)	73 332 (5.6)	78 183 (7.6)
45–54	350 170 (14.9)	211 576 (16.0)	138 593 (13.5)
55–59	274 706 (11.7)	161 826 (12.3)	112 881 (11.0)
60–64	324 416 (13.8)	188 097 (14.2)	136 319 (13.3)
65–69	326 139 (13.9)	184 344 (14.0)	141 795 (13.8)
70–74	308 156 (13.1)	171 103 (13.0)	137 053 (13.4)
75–84	467 754 (19.9)	255 577 (19.4)	212 178 (20.7)
85+	142 242 (6.1)	74 146 (5.6)	68 096 (6.6)
CKD stage (eGFR in mL/min/1.73 m^2^)			
No CKD (eGFR≥60)	934 070 (39.8)	580 871 (44.0)	353 199 (34.5)
CKD stage 3a (eGFR 45–59)	113 238 (4.8)	68 074 (5.2)	45 163 (4.4)
CKD stage 3b (eGFR 30–44)	18 435 (0.8)	10 873 (0.8)	7562 (0.7)
CKD stage 4 (eGFR 15–29)	1926 (0.1)	1036 (0.1)	890 (0.1)
Baseline CKD status absent	1 277 429 (54.5)	659 145 (49.9)	618 283 (60.3)
Comorbidities			
Diabetes mellitus	504 053 (21.5)	371 423 (28.1)	132 630 (12.9)
Ischaemic heart disease	735 949 (31.4)	437 433 (33.1)	298 516 (29.1)
Cardiac failure	152 904 (6.5)	116 449 (8.8)	36 456 (3.6)
Arrhythmia	281 141 (12.0)	156 555 (11.9)	124 586 (12.2)
Hypertension	2 036 050 (86.8)	1 194 641 (90.5)	841 409 (82.1)
Other antihypertensive drugs			
β blockers	764 584 (32.6)	289 190 (21.9)	475 394 (46.4)
Calcium channel blockers	732 628 (31.2)	331 429 (25.1)	401 199 (39.1)
Thiazides	742 535 (31.7)	328 679 (24.9)	413 855 (40.4)
Non-thiazide diuretic drugs			
Loop diuretics	155 911 (6.6)	118 565 (9.0)	37 346 (3.6)
Potassium-sparing diuretics	42 047 (1.8)	25 015 (1.9)	17 033 (1.7)
Ethnicity			
White	982 377 (41.9)	569 946 (43.2)	412 431 (40.2)
South Asian	26 933 (1.1)	17 647 (1.3)	9286 (0.9)
Black	148 301 (0.6)	6723 (0.5)	8108 (0.8)
Other	7832 (0.3)	4792 (0.4)	3041 (0.3)
Mixed heritage	2553 (0.1)	1490 (0.1)	1063 (0.1)
Not stated or missing	1 310 572 (55.9)	719 403 (54.5)	591 169 (57.7)
Calendar period			
1997–2000	93 628 (4.0)	26 446 (2.0)	67 182 (6.6)
2001–2004	418 412 (17.8)	170 465 (12.9)	247 947 (24.2)
2005–2008	742 558 (31.7)	422 466 (32.0)	320 092 (31.2)
2009–2011	646 221 (27.6)	416 685 (31.6)	229 535 (22.4)
2012–2014	444 280 (18.9)	283 938 (21.5)	160 342 (15.6)

Data are person years unless otherwise stated. Numbers in brackets are column percentages unless otherwise specified.

*Note that numbers exposed to antihypertensive treatment regimens including an ACEI/ARB and excluding an ACEI/ARB do not total the whole cohort number as individuals may be included in both columns.

ACEI/ARB, ACE inhibitors inhibitor/angiotensin receptor blocker; CKD, chronic kidney disease; eGFR, estimated glomerular filtration rate.

### Association of ACEI or ARB prescription with rate of AKI

**Figure 2:**
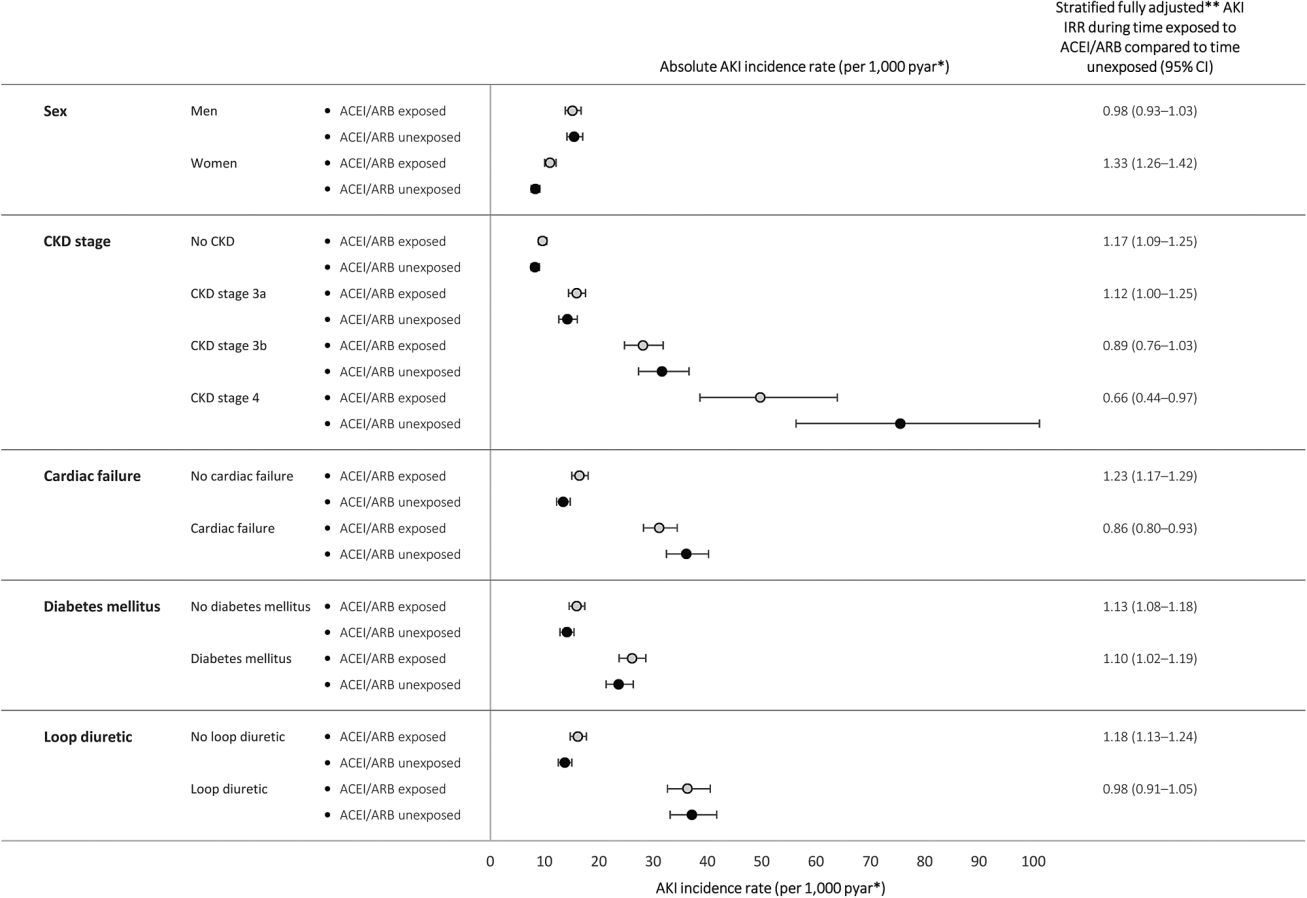
Modelled rates* of AKI (during the calendar period 2012–2014) per 1000 person years at risk for AKI during time exposed to antihypertensive treatment including ACEI/ARB compared to time exposed to antihypertensive treatment excluding ACEI/ARB, stratified by characteristics and comorbidities. *Absolute rates (unless otherwise stated) are for men, aged 75–84, with CKD stage 3a, and no comorbidities—chosen as a large, clinically important, high-risk group. **Adjusted using Poisson regression for age, sex, chronic comorbidities (CKD, hypertension, diabetes mellitus, cardiac failure, ischaemic heart disease and arrhythmia), time exposed to other antihypertensive drugs (β blockers, calcium channel blockers and thiazides), time exposed to loop and potassium-sparing diuretics and calendar period. AKI, acute kidney injury; ACEI/ARB, ACE inhibitors inhibitor/angiotensin receptor blocker; pyar, Person years at risk; CKD, chronic kidney disease; ACEI/ARB exposed, antihypertensive treatment including ACEI/ARB; ACEI/ARB unexposed, antihypertensive treatment excluding ACEI/ARB; IRR, incidence rate ratio.

The association between covariates including age and comorbidities is shown in online supplementary table S2. In the fully adjusted model, age above 70 years, baseline CKD stage 3B and above, loop diuretic treatment and cardiac failure were all associated with a greater than doubling of AKI risk. Over the whole study period, the age and sex adjusted incidence RR for first AKI comparing time exposed to antihypertensive treatment including an ACEI/ARB to that excluding an ACEI/ARB was 1.69 (95% CI 1.63 to 1.76), which fell to 1.12 (95% CI 1.07 to 1.17) after full adjustment (see online supplementary table S2). Further adjustment for lifestyle covariates and socioeconomic status made marginal difference to all results (see online supplementary table S3). Among subgroups with the highest absolute rates of AKI such as those with cardiac failure and CKD stage 4, there was no measurable association (or an apparent protective effect) of AKI with ACEI/ARB treatment (figure 2).

### Sensitivity analyses

Inclusion of only those with known baseline CKD stage, adjustment for ethnicity and varying the way that AKI was defined from ICD-10 coding made minimal differences to the RR for AKI comparing time exposed to antihypertensive treatment including an ACEI/ARB to that excluding an ACEI/ARB (see online supplementary table S3).

### Interaction between diuretics and ACEI/ARB treatment

There was an interaction between loop diuretics and ACEI/ARB treatment; there was no apparent increase in risk of AKI associated with ACEI/ARB exposure during periods of treatment with loop diuretic. Among people exposed to loop diuretics, the RR for AKI during time exposed to treatment including an ACEI/ARB compared to that excluding an ACEI/ARB was 0.98 (95% CI 0.91 to 1.24), whereas among those not requiring loop diuretics the RR was 1.18 (95% CI 1.13 to 1.24) (p<0.001). Absolute rates of AKI stratified by ACEI/ARB treatment and comorbidity, modelled with inclusion of the interaction term, highlight the higher rates of AKI among people taking loop diuretics within each stratum (see online supplementary table S4 and figure S4). There was no evidence for an interaction between potassium-sparing diuretics and ACEI/ARB treatment (RR for AKI among those prescribed potassium-sparing diuretics during time exposed to ACEI/ARB compared to time unexposed 1.09 (95% CI 0.96 to 1.24), whereas among those not requiring potassium-sparing diuretics, the RR was 1.12 (95% CI 1.08 to 1.17: p=0.667)).

### Impact of change in renal function on rate of AKI

When renal function was time updated, the RR for AKI during time exposed to antihypertensive treatment including and excluding an ACEI/ARB was attenuated to 1.02 (95% CI 0.98 to 1.07) (see online supplementary table S2). Among people who developed AKI, the median number of days between last measurement of eGFR and admission with the AKI episode was 116 days (IQR 44–258 days).

### Rate of AKI in users of ACEI compared to ARB, or both combined

Compared to time not exposed to either drug, exposure to an ACEI was associated with an adjusted RR for AKI of 1.14 (95% CI 1.09 to 1.19), while exposure to an ARB was associated with a RR of 1.06 (95% CI 1.00 to 1.12). Dual therapy with an ACEI and an ARB was associated with nearly twice the rate of AKI compared to time unexposed to either drug (RR 1.83 95% CI 1.53 to 2.17).

## Discussion

Among antihypertensive users, we found a 12% (95% CI 1.07 to 1.17) increase in the rate of AKI during time exposed to ACEI/ARB compared to time unexposed (after adjustment for comorbidities, additional drug exposure and calendar period). However, this relative risk varied markedly among different subgroups and was highest among those with the lowest absolute risk of AKI. There was no evidence of increased AKI risk for ACEI/ARB users among those at greatest absolute risk of AKI (eg, those with comorbidities or those also prescribed loop diuretics). Adjustment for most recent renal function further attenuated the risk of AKI due to ACEI/ARB exposure. We have shown that treatment with ACEI is associated with a similar magnitude of risk of AKI as ARB, but there is a near doubling of risk of AKI during time exposed to ACEI and ARB. In addition to examining the effect of antihypertensives on AKI risk, we have calculated absolute rates of AKI in a general population cohort and the impact of important comorbidities and age on these rates.

To illustrate our results, it is useful to consider the number of cases of AKI associated with ACEI/ARB use within different subgroups. Assuming that differences in AKI rates were directly attributable to ACEI/ARB exposure, in a low risk group—such as those with normal renal function—despite a 17% increase in relative risk of AKI during ACEI/ARB treatment, for 1000 people, removal of the drug would reduce the number of AKI cases from 10 to 8 per year. In contrast, in a group at high absolute risk of AKI—such as those with cardiac failure treated with loop diuretics—exposure to ACEI/ARB has minimal impact. Among 1000 such people, there are 76 cases of AKI in those treated with ACEI/ARB and 78 cases among those not treated.

### Strengths and limitations

This is the largest study of this topic to date; it examines an inclusive population-based cohort from primary care and reflects routine clinical use of these medications. By comparing ACEI/ARB use to other antihypertensives, we were able to reduce confounding by indication compared to previous case–control studies. We were able to clearly define and adjust for covariates including renal function prior to starting the medication. The time-updated analysis reduced residual confounding, while restriction to only incident users reduced adherence bias.

However, there are a number of important limitations. Our assessment of drug exposure was based on prescriptions so we cannot be certain that people prescribed the drug were taking the medication; importantly we were unable to take into account any temporary discontinuation in medication use during acute illness or hospitalisation. We did not have inpatient biochemical data so could only use ICD-10 coding to define AKI. Therefore, we have captured only a proportion of the cases defined by current biochemical definitions of AKI (although this includes a greater proportion of more severe cases^34^ ^35^) and we were not able to grade the severity of AKI. We examined first episode of AKI only. We cannot be certain that AKI was present at the time of hospital admission or developed while in hospital; although we have conducted several sensitivity analyses to investigate the impact of varying our AKI definition in an attempt to address this. It is possible that there is bias in classification of the outcome. For example, due to awareness of an association, hospital staff may be more likely to recognise and code AKI in patients taking ACEI/ARB. Alternately, patients taking these drugs may have more frequent monitoring of renal function and therefore be more likely to have AKI detected. However, these sources of bias would lead to an overestimate of the association between ACEI/ARB and AKI. We did not examine the additional effects of NSAIDs because these have been examined in previous studies.^6–8^ There is limited and selective data on proteinuria from primary care records so we were not able to adjust for this potentially important covariate. Finally, this study is limited to NHS patients in England, which may restrict its generalisability.

### Comparison to other studies

Previous high-quality evidence regarding the association between ACEI/ARB and AKI is scarce. Estimates of the increase in AKI risk associated with use of ACEI/ARB from randomised trials are limited.^5^ Many commonly cited observational studies are cross-sectional or address the risk of AKI in relation to specific diseases or interventions.^8–10^ Two recent nested case–control studies using UK primary care data reported only relative risks for AKI among users of NSAIDS in addition to ACEI/ARB and diuretics.^6^ ^7^ Only one population-based study has examined the relative risk of AKI among ACEI/ARB users compared to non-users. This study, despite limited data quality, found similar results to our own with a fully adjusted OR of 1.11 (95% CI 1.02 to 1.20) comparing those prescribed and not prescribed ACEI/ARB.^36^

However, strong evidence does exist in relation to the risks of AKI associated with combined ACEI and ARB therapy. Here, recent clinical trials using this regime have reported HRs for renal adverse events ranging from 1.20 (95% CI 0.96 to 1.50) to 2.19 (95% CI 1.13 to 4.22), compared to single agent treatment alone.^12^ ^13^ Our results, showing a near doubling of rate for AKI with dual blockade, are similar to these findings and strongly support the validity of our study.

Finally, similar to other recent studies, we show that the rate of AKI detected by ICD-10 coding has increased markedly over the time period of this study. This is well documented and likely to be multifactorial, attributable to better hospital coding, increased recognition of AKI and possibly a true increase in incidence.^37^

### Possible explanations and implications for clinicians and policymakers

Although surprising, we believe that this study has provided the most accurate estimates of the strength of the association between ACEI/ARB use and AKI to date. One alternate explanation for the small effect size is that we have compared time exposed to ACEI/ARB to other antihypertensives, with no untreated comparison group. While this design reduces confounding by indication, it is possible that all antihypertensives increase AKI risk during acute illness. In addition, among those at the highest absolute risk of AKI (eg, those with additional comorbidities), we found rates of AKI for ACEI/ARB users were lower than those for non-users. The probable explanation for these findings is that, in patients with multiple comorbidities that are indications for ACEI/ARB treatment, not being treated with ACEI or ARB is a marker of unmeasured poor health status or frailty. For example, an individual may have stopped ACEI/ARB treatment when they became unwell, perhaps due to worsening renal function, and the reason for stopping ACEI/ARB places them at higher risk of AKI during subsequent follow-up. This is likely to have attenuated the estimate of the strength of association between ACEI/ARB and AKI over the whole study population, although the proportion of patients with multiple comorbidities is small. Finally, our results do not exclude that AKI among users of ACEI/ARB is more severe compared to users of other antihypertensives. However, even when the AKI definition was restricted to code positions representing the primary diagnosis, ACEI/ARB treatment was only associated with a 21% (95% CI 11% to 33%) increase in AKI rate.

We have also shown that patients taking loop diuretics have higher rates of AKI than similar patients not prescribed the drugs. This may be causal, due to salt and water depletion during acute illness, or additional loop diuretic treatment may be a marker of severity of comorbidities. We anticipated that pharmacological interaction between loop diuretics and ACEI/ARB would be associated with an increased risk of AKI compared to treatment with loop diuretics alone but we have shown the converse. Again, this is likely to be explained by confounding by underlying health status where the most severely unwell patients at highest risk of AKI are not treated with diuretics and ACEI/ARB. Finally, we have shown that when adjusted for most recent renal function, there was no measurable association between ACEI/ARB use and AKI. The findings of this analysis have to be considered in light of possible misclassification of CKD stage by renal function measured during an AKI episode, although we excluded all measurements of kidney function that occurred before of an admission with AKI.

Treatment with ACEI and ARB are widely believed to be risk factors for AKI, particularly during acute illness. This underlies the ‘sick-day rules’ recommendation for patients to stop taking these drugs when they become acutely unwell with symptoms of gastroenteritis or fever.^17^ This study was not designed to examine the effect of temporary cessation of these drugs on the development of AKI. However, we have demonstrated that patient comorbidities are much stronger risk factors for the development of AKI than these drugs and that there is no measurable effect of the drugs among those at highest risk of AKI. This reinforces the importance of assessing overall risk of AKI in planning potential interventions aimed at lowering hospital admissions with AKI. Patients with multiple risk factors but not taking ACEI/ARB may be those who would benefit most from close review of fluid balance and intensive monitoring during acute illness, but they may be overlooked if the clinical focus is on drug cessation.

## Conclusions

Our results show that treatment with ACEI/ARB appears to be associated with only a small increase in AKI risk while patient characteristics (such as age and comorbidities) are much more strongly associated with the rate of AKI. While people may benefit from optimised medicines management during acute illness, our results suggest that these interventions should be targeted at individuals at highest risk of AKI rather than focussing on users of ACEI/ARB.
