# Supplementary material for: Prescription of renin–angiotensin system blockers and risk of acute kidney injury: a population-based cohort study
Source: BMJ Open. 2016 Dec 21;6(12):e012690. doi: 10.1136/bmjopen-2016-012690 (PMC5223684; doi:10.1136/bmjopen-2016-012690)
Supplement: supplementary data [file bmjopen-2016-012690supp.pdf]

Text S1: Antihypertensive drug exposure defined by multiple binary indicators

Rather than a single variable representing time exposed to a single class of antihypertensive, we used four time-updating, binary indicator variables to indicate antihypertensive exposure status. Defining exposure status in this way allowed patients to be exposed to more than one class of antihypertensive at a time. Each indicator variable identified whether the associated period of time at risk was exposed (1) or unexposed (0) to a specific class of antihypertensive. Figure S1 illustrates how the indicator variables would be assigned in three example scenarios. In scenario one, the patient remains exposed to only one agent for the duration of the study. In scenario two, a second antihypertensive is added. In scenario three, the patient switches from one class of drug to a different class of drug.

Figure S1: Assignment of time-varying exposure status during follow-up using multiple binary indicator exposure variables under three example scenarios.

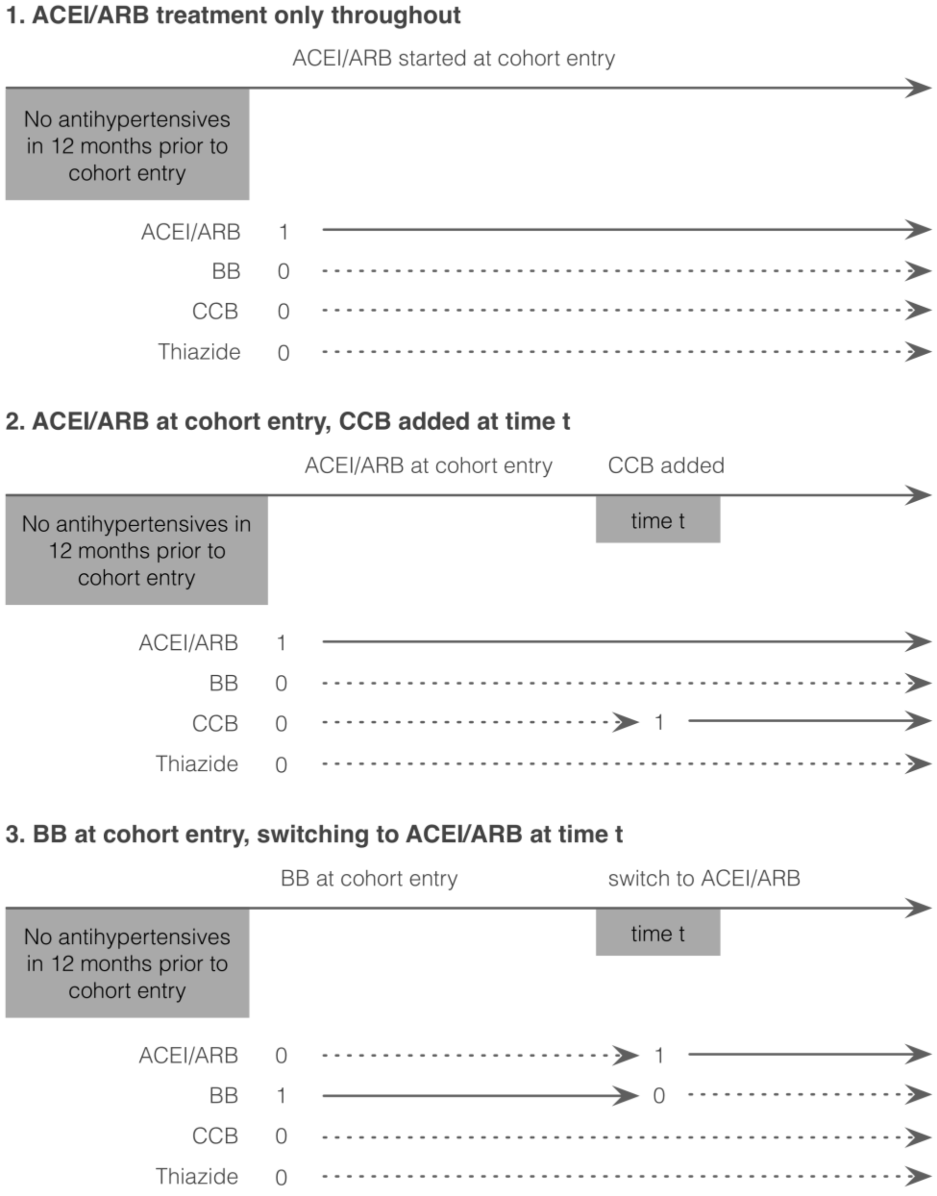

0=indicator variable 'switched off' (drug not prescribed); 1=indicator variable 'switched on' (drug prescribed).
ACEI/ARB: Angiotensin converting enzyme inhibitor/angiotensin receptor blocker
BB: Beta-blocker
CCB: Calcium channel blocker

Table S1. Definition of acute kidney injury: ICD-10 codes used to define of acute kidney injury in HES data.

ICD-10 code	Clinical term	% of cases of AKI cases defined by code
N14.1	Nephropathy induced by other drugs, medicaments and biological substances	0.12
N14.2	Nephropathy induced by unspecified drug, medicament or biological substance	0.03
N17.0	Acute renal failure with tubular necrosis	0.78
N17.1	Acute renal failure with acute cortical necrosis	0.04
N17.2	Acute renal failure with medullary necrosis	0.03
N17.8	Other acute renal failure	0.28
N17.9	Acute renal failure, unspecified	70.14
N19	Unspecified kidney failure	25.31
N99.0	Post procedural renal failure	1.29
R34	Anuria and oliguria	0.90
R94.4	Abnormal results of kidney function studies	0.99

Text S2. Definition of end-stage renal disease

ESRD was defined using hospital and primary care morbidity coding, and hospital procedure coding as the presence of:

i) An ESRD morbidity code.
ii) A code for renal transplant.
iii) A code for peritoneal or haemodialysis.
iv) Stage 5 chronic kidney disease (identified using morbidity coding and estimated glomerular filtration rate (eGFR).
v) Stage 4 CKD with a fistula, suggesting ESRD is anticipated.

Figure S2. Simplified directed acyclic graph (DAG) illustrating implicitly assumed causal structure underlying our adjusted models.

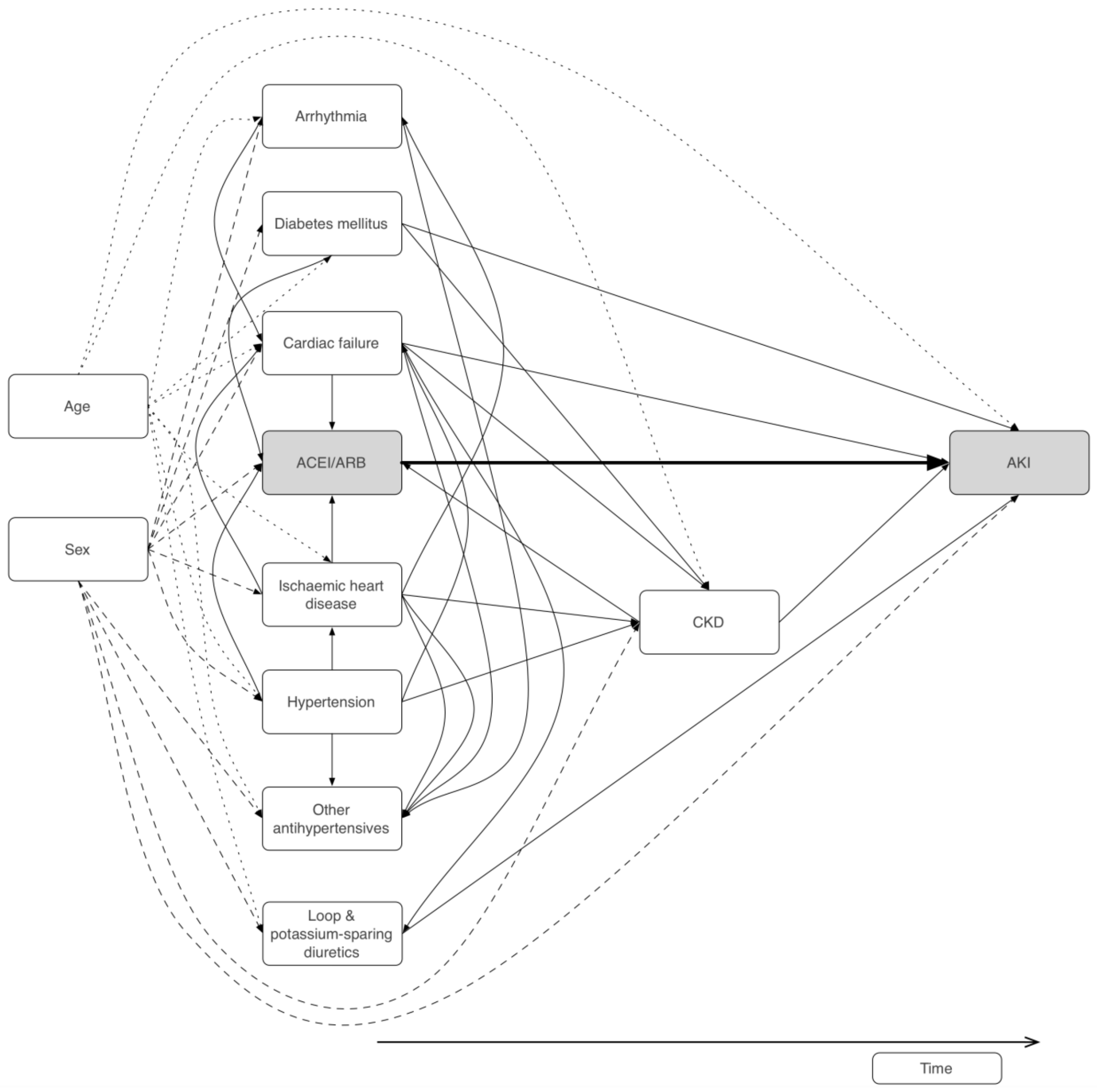

➔ Represents causal path of interest

Under this assumed framework, adjusting for all measured variables blocks confounding pathways.

AKI: Acute kidney injury
ACEI/ARB: Angiotensin converting enzyme inhibitor/angiotensin receptor blocker
CKD: Chronic kidney disease

Table S2. Age and sex, and fully adjusted* incidence rate ratios (95% CIs) for AKI in sequentially adjusted models.

	AKI incidence rate ratio (95% CI)		
	Age & sex adjusted	Fully adjusted*	Fully adjusted* with CKD stage as a time-updated variable
	n=570,445	n=570,445	n=570,445
**Exposure of interest**			
ACEI/ARB	1.69 (1.63–1.76)	1.12 (1.07–1.17)	1.02 (0.98–1.07)
**Sex**			
Female	0.61 (0.59–0.63)	0.66 (0.64–0.68)	0.63 (0.61–0.65)
**Age**			
18–44	reference	reference	reference
45–54	0.85 (0.74–0.97)	0.81 (0.71–0.93)	0.89 (0.77–1.02)
55–59	1.08 (0.94–1.23)	0.97 (0.85–1.12)	1.07 (0.93–1.22)
60–64	1.41 (1.24–1.61)	1.18 (1.03–1.35)	1.25 (1.09–1.42)
65–69	2.11 (1.86–2.38)	1.59 (1.41–1.80)	1.55 (1.36–1.76)
70–74	3.15 (2.80–3.55)	2.17 (1.92–2.45)	1.84 (1.62–2.09)
75–84	6.03 (5.37–6.78)	3.34 (2.96–3.76)	2.26 (2.00–2.56)
85+	15.06 (13.34–17.01)	5.54 (4.87–6.30)	2.98 (2.61–3.41)
**Comorbidity**			
CKD stage			
No CKD	N/A	reference	reference
CKD stage 3a	N/A	1.67 (1.57–1.77)	2.60 (2.46–2.75)
CKD stage 3b	N/A	3.19 (2.91–3.51)	5.77 (5.42–6.15)
CKD stage 4	N/A	6.69 (5.57–8.03)	13.44 (12.45–14.51)
Baseline CKD status absent	N/A	1.18 (1.13–1.23)	1.90 (1.74–2.08)
Diabetes mellitus	N/A	1.65 (1.59–1.72)	1.52 (1.46–1.58)
Ischaemic heart disease	N/A	1.14 (1.09–1.19)	1.12 (1.07–1.17)
Cardiac failure	N/A	2.09 (1.97–2.20)	1.92 (1.82–2.03)
Arrhythmia	N/A	1.42 (1.36–1.48)	1.45 (1.38–1.51)
Hypertension	N/A	1.09 (1.04–1.15)	1.01 (0.96–1.06)
**Other antihypertensive drugs**			
Beta-blocker	N/A	0.99 (0.95–1.03)	0.90 (0.87–0.94)
Calcium channel blocker	N/A	0.97 (0.93–1.01)	0.93 (0.90–0.97)
Thiazide	N/A	0.99 (0.95–1.04)	0.91 (0.87–0.96)
**Non-thiazide diuretics**			
Loop	N/A	2.36 (2.23–2.50)	1.77 (1.67–1.88)
Potassium sparing	N/A	1.86 (1.74–1.98)	1.67 (1.56–1.79)
**Calendar period**			
1997–2000	N/A	reference	reference
2001–2004	N/A	1.17 (1.03–1.34)	1.14 (0.99–1.32)
2005–2008	N/A	1.62 (1.42–1.85)	1.61 (1.39–1.86)
2009–2011	N/A	2.26 (1.99–2.57)	2.38 (2.06–2.75)
2012–2014	N/A	3.29 (2.89–3.74)	3.62 (3.12–4.19)

*Adjusted using Poisson regression for: age, sex, chronic comorbidities (CKD, hypertension, diabetes mellitus, cardiac failure, ischaemic heart disease, and arrhythmia), time exposed to other antihypertensive drugs (beta-blockers, calcium channel blockers and thiazides), time exposed to loop and potassium-sparing diuretics, and calendar period
AKI: Acute kidney injury
ACEI/ARB: Angiotensin converting enzyme inhibitor/angiotensin receptor blocker
CKD: Chronic kidney disease
eGFR: Estimated glomerular filtration rate

Table S3. Incidence rate ratios (95% CIs) for AKI during time exposed to ACEI/ARB compared to time exposed to other antihypertensives in the main analysis and additional sensitivity analyses.

	Number of individuals	Number of AKI cases identified	AKI incidence rate ratio (95% CI) for ACEI/ARB therapy (compared to time exposed to other antihypertensives)	
			Age and sex adjusted	Fully adjusted*
**Main analysis:** AKI defined using all ICD-10 codes in any position and any episode within 28 days of the start of a hospital admission.	570,445	14,907	1.69 (1.63–1.76)	1.12 (1.07–1.17)
AKI defined using only code N17 in any position and any episode within 28 days of the start of a hospital admission.	570,445	11,174	1.76 (1.68–1.84)	1.12 (1.06–1.17)
AKI defined using all ICD-10 codes in any diagnostic position in the 1st or 2nd episode for episodes that occur within 7 days of admission.	570,445	14,377	1.70 (1.64–1.77)	1.13 (1.09–1.18)
AKI defined using all ICD-10 codes in the 1st diagnostic position of the 1st or 2nd episode for episodes that start within 7 days of admission.	570,445	3,009	1.83 (1.69–1.97)	1.21 (1.11–1.33)
Restricted to those with known baseline CKD status.	268,456	7,312	1.56 (1.47–1.65)	1.09 (1.02–1.16)
Including time-updated CKD stage rather than baseline CKD stage.	570,445	14,907	1.69 (1.63–1.76)	1.02 (0.98–1.07)
Additionally adjusting for smoking status, alcohol intake, and BMI.	485,139	12,583	N/A	1.17 (1.12–1.22)
Additionally adjusting for smoking status, alcohol intake, BMI, and socioeconomic status.	484,525	12,559	N/A	1.18 (1.12–1.23)
Restricted to those entering the cohort from 2006 with known ethnicity.**	132,847	1,944	1.37 (1.24–1.52)	1.07 (0.95–1.21)

*Adjusted using Poisson regression for: age, sex, chronic comorbidities (CKD, hypertension, diabetes mellitus, cardiac failure, ischaemic heart disease, and arrhythmia), time exposed to other antihypertensive drugs (beta-blockers, calcium channel blockers and thiazides), time exposed to loop and potassium sparing diuretics, and calendar period.
**Additionally adjusted for ethnicity and with race used to calculate eGFR
AKI: Acute kidney injury
ACEI/ARB: Angiotensin converting enzyme inhibitor/angiotensin receptor blocker
CKD: Chronic kidney disease
eGFR: Estimated glomerular filtration rate

Table S4. Descriptive table of modelled rates of AKI (during the calendar period 2012–2014) per 1000 person years at risk stratified by characteristics and comorbidities. This model includes an interaction term for loop diuretic use.

	**Rate of AKI in ACEI/ARB exposed per 1000 pyar***		**Rate of AKI in ACEI/ARB unexposed per 1000 pyar***		**Absolute rate difference per 1000 pyar**	
	On loop	Not on loop	On loop	Not on loop	On loop	Not on loop
**Sex (rates for each sex aged 75-84 with CKD stage 3A and no comorbidities)**						
Men	36.3	16.1	37.1	13.7	-0.8	2.4
Women	24.0	10.6	24.5	9.0	-0.5	1.6
**Age (rates for each age group for men with CKD stage 3A and no comorbidities)**						
18–44	10.9	4.8	11.1	4.1	-0.2	0.7
45–54	8.8	3.9	9.0	3.3	-0.2	0.6
55–59	10.6	4.7	10.8	4.0	-0.2	0.7
60–64	12.8	5.7	13.1	4.8	-0.3	0.9
65–69	17.3	7.7	17.7	6.5	-0.4	1.2
70–74	23.6	10.5	24.1	8.9	-0.5	1.6
75–84	36.3	16.1	37.1	13.7	-0.8	2.4
85+	60.1	26.7	61.5	22.6	-1.4	4.1
**CKD stage (rates for each CKD stage for men with no comorbidities)**						
No CKD (eGFR >=60)	21.8	9.7	22.3	8.2	-0.5	1.5
CKD stage 3a (eGFR 45–59)	36.3	16.1	37.1	13.7	-0.8	2.4
CKD stage 3b (eGFR 30–44)	69.3	30.8	70.8	26.1	-1.5	4.7
CKD stage 4 (eGFR 15–29)	144.3	64.1	147.5	54.3	-3.2	9.8
**Comorbidity (rates for men aged 75-84 with CKD stage 3A and only the specified comorbidity)**						
Diabetes mellitus	59.9	26.6	61.2	22.5	-1.3	4.1
Ischaemic heart disease	41.3	18.4	42.2	15.5	-0.9	2.9
Cardiac failure	76.3	33.9	78.0	28.7	-1.7	5.2
**Risk group**						
Lowest risk group [1]	4.3	1.9	4.4	1.6	-0.1	0.3
Highest risk group [2]	648.7	288.3	663.1	244.1	-14.4	44.2

[1] Low risk: women aged 18-44 without CKD and no comorbidities
[2] High risk: men aged 85+ with CKD stage 4 and all of: ischaemic heart disease, cardiac failure and diabetes mellitus.

*Adjusted using Poisson regression for: age, sex, chronic comorbidities (CKD, hypertension, diabetes mellitus, cardiac failure, ischaemic heart disease, and arrhythmia), time exposed to other antihypertensive drugs (beta-blockers, calcium channel blockers and thiazides), time exposed to loop and potassium sparing diuretic, and calendar period. Also includes an interaction term for loop diuretic use.

Figure S4. Modelled rates of AKI (during the calendar period 2012–2014) per 1,000 person years at risk stratified by characteristics and comorbidities. This model includes an interaction term for loop diuretic use.

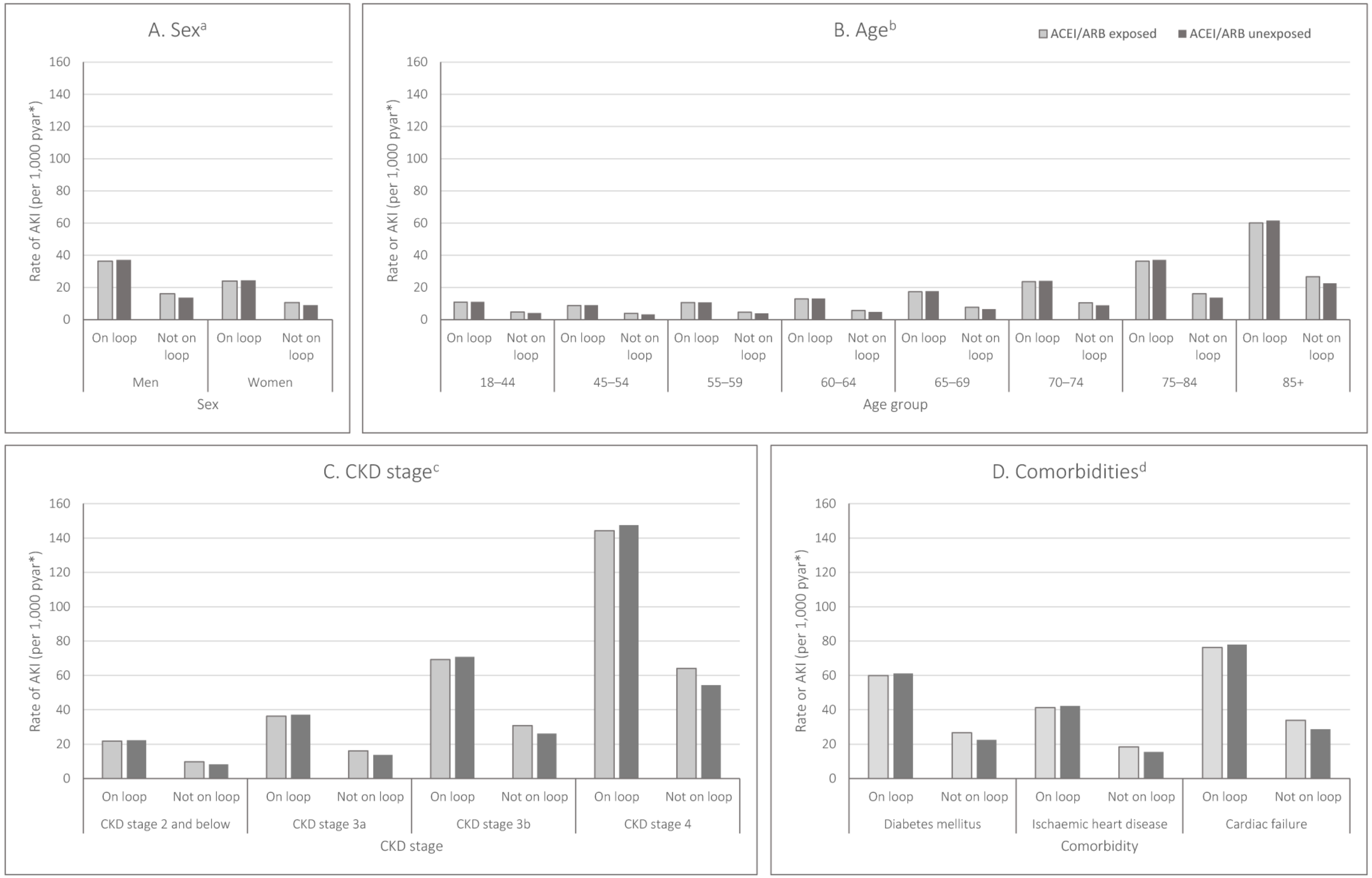

*Adjusted using Poisson regression for: age, sex, chronic comorbidities (CKD, hypertension, diabetes mellitus, cardiac failure, ischaemic heart disease, and arrhythmia), time exposed to other antihypertensive drugs (beta-blockers, calcium channel blockers and thiazides), time exposed to loop and potassium sparing diuretics, and calendar period. Also includes an interaction term for loop diuretic use.
Absolute rates (unless otherwise stated) are for men, aged 75–84, with CKD stage 3a, and no comorbidities – chosen as a large, clinically important, high risk group.
[a]rates for each sex aged 75-84 with CKD stage 3A and no comorbidities.
[b]rates for each age group for men with CKD stage 3A and no comorbidities.
[c]rates for each CKD stage for men with no comorbidities.
[d]rates for men aged 75-84 with CKD stage 3A and only the specified comorbidity.
ACEI/ARB: Angiotensin converting enzyme inhibitor/angiotensin receptor blocker.
AKI: Acute kidney injury
pyar: Person years at risk
CKD: Chronic kidney disease
